# A new strategy to measure intercellular adhesion forces in mature cell-cell contacts

**DOI:** 10.1038/srep46152

**Published:** 2017-04-10

**Authors:** Ana Sancho, Ine Vandersmissen, Sander Craps, Aernout Luttun, Jürgen Groll

**Affiliations:** 1 Department of Functional Materials in Medicine and Dentistry and Bavarian Polymer Institute (BPI), University of Würzburg, 97070 Würzburg, Germany; 2 Department of Cardiovascular Sciences, Center for Molecular and Vascular Biology, KU Leuven, 3000 Leuven, Belgium

## Abstract

Intercellular adhesion plays a major role in tissue development and homeostasis. Yet, technologies to measure mature cell-cell contacts are not available. We introduce a methodology based on fluidic probe force microscopy to assess cell-cell adhesion forces after formation of mature intercellular contacts in cell monolayers. With this method we quantify that L929 fibroblasts exhibit negligible cell-cell adhesion in monolayers whereas human endothelial cells from the umbilical artery (HUAECs) exert strong intercellular adhesion forces per cell. We use a new *in vitro* model based on the overexpression of Muscle Segment Homeobox 1 (MSX1) to induce Endothelial-to-Mesenchymal Transition (EndMT), a process involved in cardiovascular development and disease. We reveal how intercellular adhesion forces in monolayer decrease significantly at an early stage of EndMT and we show that cells undergo stiffening and flattening at this stage. This new biomechanical insight complements and expands the established standard biomolecular analyses. Our study thus introduces a novel tool for the assessment of mature intercellular adhesion forces in a physiological setting that will be of relevance to biological processes in developmental biology, tissue regeneration and diseases like cancer and fibrosis.

Mechanobiology is an emerging and rapidly growing field of research that focuses on the role of physical forces in cellular function and on processes at the level of whole organisms such as tissue and organ development, physiology and the origin and progression of disease. On a cellular level, adhesion forces occur between cells and their surrounding matrix as well as between neighbouring cells. Cell-matrix interactions and related signalling pathways that are predominantly mediated through the integrin cell receptor family have for many years been in the focus of biophysical research^1^. Aside from the established biomolecular techniques to measure the presence and upregulation of adhesion-related molecules in the cell or on the cell membrane, one of the most commonly used techniques for the quantification of cell-matrix adhesion forces is Atomic Force Microscopy (AFM)^2^, where a cantilever deflects proportionally to the cell detachment force. The most common approach consists of immobilising a cell at the cantilever tip and using it as a measuring probe, by setting this cell in contact with the surfaces under study for a defined time. In order to achieve a firm adhesion of the cell at the cantilever, the latter needs to be functionalised first by means of lectins, streptavidin or proteins of the extracellular matrix (ECM). Nevertheless, on one hand, these strategies can alter the functional state or integrin distribution of cells and provide a biased result^3^,^4^, on the other hand, they are limited to cell-substrate contact times of several seconds, and thus are restricted to early cell adhesion events^5^. Another alternative consists of bringing a protein-coated cantilever onto a cell that is firmly adhered to the substrate under study, keeping it in contact with the cell for several minutes and then retracting the cantilever. As in the previous case, and depending on the functionalisation method and the biomaterial under study, this strategy can also bias cell behaviour and is also limited by the maximum detachment force that it can measure^6^. Recently, a new technology has been introduced, called FluidFM, which incorporates microfluidic probes connected to a pumping system^7^. With this technology, cells are immobilised at the cantilever tip by directly applying suctioning pressure on the cell, without any biomolecular functionalisation. Therefore, on one hand, it provides very high immobilisation forces, allowing the detachment of cells from substrates to which they are firmly adhered, and even from highly structured substrates^8^,^9^. On the other hand, it does not alter the functional state of cells or the distribution of integrins as occurs when functionalising with lectins or proteins^10^.

Beyond cell-substrate interaction, cell cohesion plays a crucial role in many biological processes, such as embryogenesis, morphogenesis and malignancy^11^,^12^. In the context of tissue engineering this is also relevant in processes like tissue spreading^13^ and cell condensation for the formation of organ buds^14^. One example of a process that in its early phase is characterised by a change in cell-cell adhesion forces is Epithelial-to-Mesenchymal Transition (EMT). A particular case of EMT is Endothelial-to-Mesenchymal Transition (EndMT), which is associated with the acquisition of mesenchymal and stem cell-like characteristics by the endothelium that lines the inside of the cardiovascular system. This process is a crucial mediator of endocardial cushion formation and subsequent cardiac valve development^15^, cancer progression^16^, fibrodysplasia ossificans progressiva^17^ and renal, pulmonary and cardiac fibrosis^18^. While extensive work has been completed to identify the biomolecular drivers of EndMT, direct measurement of the biomechanical forces involved remains unexplored^19^.

Cell-cell contacts and interactions are routinely analysed via biochemical markers such as selectins and cadherins. As indirect assessment techniques of these interactions, molecular tension sensors based on Förster resonance energy transfer (FRET)^20^ and Monolayer Stress Microscopy (MSM)^21^ have been recently established. MSM is based on Traction Force Microscopy (TFM) and the stress balance within a cell monolayer, and has mostly been employed in the context of collective migration and the transmission of tension throughout the monolayer^22^. Yet, it does not inform about the robustness of those intercellular adhesions or the maximum pulling force they are capable to endure. For the direct measurement of cell-cell adhesion forces, the most commonly used techniques are AFM and Dual Micropipette Aspiration (DPA)^20^,^23^. They are both based on the detachment forces measured as a response to an external pulling stimulus. In the case of DPA, two suspended cells are pressed gently against each other for a defined time; then, they are separated again and the detachment force is quantified. In the case of AFM, the most traditional way to measure cell-cell adhesion forces consists of immobilising a cell at the tip of a functionalised cantilever, as explained above. The immobilised cell is then pressed against cells in monolayer for several seconds and then separated again. The detachment force, and events during retraction are recorded and quantified^24^. While these techniques provide abundant information on adhesion events at the molecular level, they are restricted to work only with cells at the initial stage of the adhesion process^25^, before they establish mature intercellular junctions^26^. In this framework, the need for the quantification of the intercellular adhesion forces in a physiological setting that is most representative of their natural context, such as a cell monolayer, remains unanswered.

Here, we present a novel methodology to measure the intercellular adhesion forces exerted by cells in monolayer, after firm adhesion to the substrate and formation of mature intercellular junctions. We use FluidFM technology to completely detach cells from a monolayer and quantify the corresponding adhesion force. By comparing these forces with mature cell-substrate adhesion in subconfluent conditions, we show that L929 fibroblasts show negligible cell-cell adhesion forces, whereas human endothelial cells from the umbilical artery (HUAECs) exert strong intercellular forces. As a direct application of the quantification methodology to a biologically relevant case, we then focused our study on EndMT. Using a new *in vitro* model for the induction of EndMT, we quantify cellular adhesion and show that intercellular forces in monolayer decrease at early stages of EndMT, while cells become stiffer and thinner. These results reinforce and complement the biomolecular analyses and expand the knowledge and understanding of the biological process.

## Results

### Quantification of cell-substrate and cell-cell adhesion forces of adherent cells

**Figure 1:**
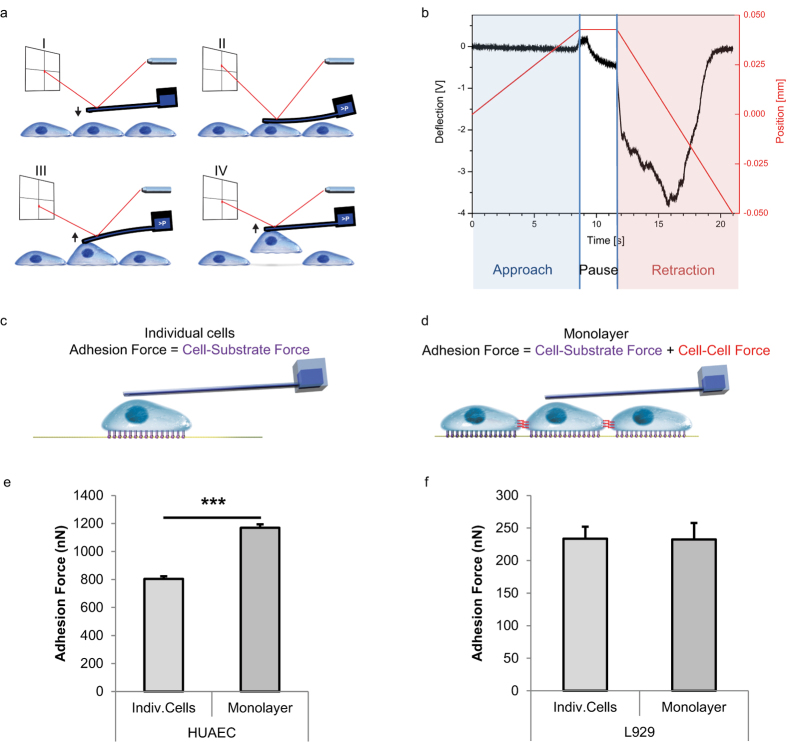
Measurement of cell-substrate and cell-cell adhesion forces. (**a**) Diagram showing the entire cycle of cell detachment, represented in four sections: I, approach; II, reached set point and immobilisation of the cell at the cantilever by fluidic pressure; III, retraction and pulling of the cell; IV, complete detachment of the cell. (**b**) Electrical signal of the cantilever deflection recorded at the photodetector and stage position during an entire cycle of cell detachment. Sections depicted in (**a**) are indicated on top. (**c**) Diagram indicating cell adhesion of individual cells. (**d**) Diagram indicating cell adhesion of cells in monolayer. (**e**,**f**) Cell adhesion forces of HUAECs (**e**) or L929 cells (**f**) as individual cells or as cells in monolayer. High cohesiveness of HUAECs is shown by the remarkable difference between single cell and monolayer conditions. Error bars represent standard error of the mean (s.e.m.) of the total number of measured cells (HUAECs: *n* = 30; L929 cells: *n* = 14), ***P = 0.0006 by Student’s *t*-test. Measurements in (**e**,**f**) were performed by cell detachment between days 1 and 3 after cell seeding; the provided values correspond to the average of the maximum detachment forces.

We have measured the cell-substrate adhesion force on glass of various cell types by Single Cell Force Spectroscopy (SCFS) using FluidFM technology incorporated into the AFM (Fig. 1a,b). Measurements were performed on “individual cells”, meaning cells that are not in direct contact with other cells, and on cells in monolayer, which are fully surrounded by other cells. Our measurements on L929 mouse fibroblasts as individual cells showed an adhesion force to the glass of 234 nN which is equal to the force exerted by 3T3 mouse fibroblasts on the same substrate measured with a traditional AFM by Weder *et al*.^27^. In the context of strongly adherent cells, we have measured HUAECs, which as individual cells showed an adhesion force of 805 nN. Moving further towards a more biologically representative context for the endothelial cells (ECs), we have cultured HUAECs in monolayer, and measured the adhesion force they exert in this tissue-like condition. HUAECs showed an average force of 1,170 nN when they are detached from a monolayer (Fig. 1e; Supplementary Video SV1). In such conditions, even values above 3,500 nN have been recorded, despite being discarded as outliers. While the force measured on individual cells represents the adhesion force between a cell and the substrate, the force measured in monolayer indicates the adhesion force of a cell to the substrate and to the surrounding cells that are in direct contact with it (Fig. 1c,d). Therefore, we propose an approach to calculate the cell-cell adhesion forces consisting on subtracting the adhesion force of individual cells from the force measured in monolayer (equation (1)). According to equation (1), our results show that HUAECs exert an average cell-cell adhesion force of 365 nN. This force value agrees with the known characteristic of arterial ECs for being highly cohesive cells^28^.


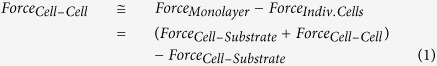


For validation of the methodology, we chose L929 cells as a known non-cohesive cell type^13^. There, we measured the adhesion force of cells in monolayer and we calculated the intercellular adhesion force according to equation (1) (Supplementary Video SV2). Measurements revealed an adhesion force of 232 nN in monolayer, which indicates negligible cell-cell adhesion forces, as expected from non-cohesive cells (Fig. 1f). The proposed methodology assumes equal cell-substrate adhesion forces of individual cells and cells in monolayer, meaning that it does not take into consideration the cross-talk between cadherins and integrins^29^, nor the reduction in the projected area of cells in monolayer. As a first approach to further support our assumption, we compared the density of vinculin-expressing focal adhesions (FAs) between HUAECs in individual cell state and HUAECs in monolayer. FAs contain integrins and are responsible for the interaction of the cell with the substrate^30^. We found no significant differences between the individual state and cells in monolayer, the number of FA per cell area being 0.038 ± 0.0004 and 0.039 ± 0.0039, respectively, with area expressed in μm^2^ (*n* = 10 cells for each condition). Yet, the lack of a precise quantification of the influence of cadherin and integrin cross-talk in cell-substrate adhesion forces is currently an unavoidable limitation of the methodology. Nevertheless, the imperceptible cell-cell adhesion forces obtained in the non-cohesive cells and the high forces obtained in the arterial ECs fulfilled the theoretical expectations. Thus, we concede that the method represents a valid approach to assess the mature intercellular adhesion forces present in a tissue-like situation.

In order to underscore and demonstrate the capabilities of this methodology for mechanobiology to study biological processes where cell-cell interactions play a relevant role, we have chosen EndMT as a case study. In several *in vivo* pathological models EndMT has been associated with the Bone Morphogenetic Protein (BMP) signalling pathway^17^,^18^,^31^. Moreover, the stimulation of BMP *in vitro* induces a phenotypic and molecular transition of ECs to mesenchymal-like cells^17^. A downstream effector and upstream mediator within the BMP-signalling cascade active in ECs is the transcription factor Muscle Segment Homeobox 1 (MSX1). It is involved in BMP-mediated EndMT in endocardial ECs during atrioventricular valve formation *in vivo*^32^,^33^,^34^. However, it remains unclear whether MSX1 induction alone is sufficient to direct EndMT of ECs in culture. In the following sections we combine the biomechanical and the biochemical characterisation performed in early stages of EndMT, where weakening of intercellular interactions are known to occur. We induce the transition by overexpression of the gene encoding MSX1 and we show that this upregulation is sufficient to induce EndMT *in vitro*. In order to validate the biological process we have repeated the biomechanical characterisation in a second clone.

### MSX1 overexpression induces molecular and morphological changes consistent with EndMT

**Figure 2:**
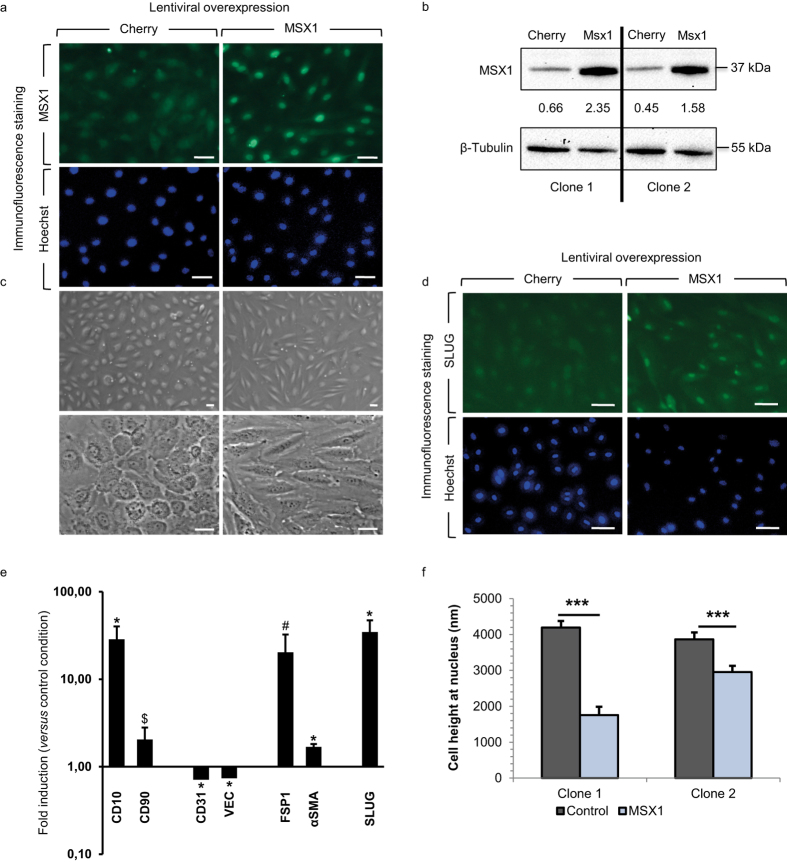
Overexpression of MSX1 causes elongation and flattening of HUAECs. (**a**) Nuclear expression of MSX1 (green) in HUAECs after lentiviral transduction with MSX1- or Cherry-encoding reporter viruses. Hoechst (blue) was used as a nuclear marker. (**b**) Western blot and corresponding densitometric quantification (normalised for ß-tubulin as loading control) showing MSX1 protein overexpression in 2 clones (see Supplementary Fig. S5 for full-length blots). (**c**) Phase contrast pictures showing the phenotypic change from cobblestone-like to spindle-like shape upon overexpression of MSX1. (**d**) Immunofluorescence staining for mesenchymal transcription factor *Slug* (green) and nuclear marker Hoechst (blue). An induction of *Slug* is observed in MSX1-overexpressing cells. Scale bars in (a,c,d): 25 μm. (**e**) Gene expression analysis by qRT-PCR. It shows a down-modulation of endothelial markers and induction of stem cell and mesenchymal markers. mRNA expression levels upon MSX1 overexpression are represented as fold induction *versus* control condition, in ‘*n*’ independent experiments, *n* = 7 for *CD90, FSP1*; *n* = 8 for *CD31, SLUG, VEC*; *n* = 9 for *αSMA, CD10*. Error bars represent the standard error of the mean (s.e.m.), ^#^*P* = 0.07, ^$^*P* = 0.1, *P < 0.05 by one sample Student’s *t*-test with null-hypothesis 1. (**f**) Height of the cells measured in two independent clones on the nuclear region by indentation with colloids using AFM with FluidFM add-on technology. MSX1-overexpressing cells are thinner than Cherry control cells. Error bars represent s.e.m. of the total amount of cells measured per condition (*n*). ***P = 7∙10^−10^ in clone 1 and ***P = 0.0008 in clone 2 by Student’s *t*-test, *n* = 20, 18, 36 and 37, with ‘*n*’ representing the number of indented cells for control and MSX1 conditions for clone 1 and 2, respectively.

We lentivirally transduced primary HUAECs with either MSX1-encoding viruses or with Cherry reporter viruses, the latter as control. We confirmed robust nuclear MSX1 overexpression by immunofluorescence staining and by Western blot (Fig. 2a,b). We also confirmed *MSX1* mRNA overexpression by quantitative (q)RT-PCR in both clones (revealing a 420-fold and 345-fold upregulation, respectively). EndMT induces a strong change in cell shape from a cobblestone EC-like to a spindle shape mesenchymal cell-like morphology^35^, which we also observed upon MSX1 overexpression (Fig. 2c). We quantitatively assessed the shape change by measuring area, perimeter, major and minor axis of cells in monolayer and calculated the corresponding aspect ratio, circularity and roundness (Table 1). Roundness decreased from 0.7 in control cells to 0.2 in MSX1-overexpressing cells; while circularity decreased from ~0.8 to 0.3, within a range of 0 to 1, where 1 indicates a perfect circular and round shape. MSX1-overexpressing cells exhibited longer major and shorter minor axes, compared with the control cells, which led to an increase in the aspect ratio from ~1.5 to 4.5 or 6. As a result, while both cell types have the same perimeter, *i*.*e*., they have the same size, MSX1-overexpressing cells showed a noticeable reduction in the projected cell area, caused by an enlarged aspect ratio. This increased aspect ratio with decreased circularity and roundness, and the reduction of the projected area while maintaining the perimeter length corroborated the abovementioned shape change characteristic of EndMT processes upon overexpression of MSX1. Another morphological change known to occur in EndMT is a reduction in cell height^36^. Therefore, we measured cell height at the nuclear region using FluidFM technology. A colloid of 10 μm in diameter was immobilised at the tip of the cantilever and indentations were performed on the nucleus of cells and on glass. Cell height was calculated as the difference between the cell and the glass contact points. These measurements confirmed that MSX1-overexpressing cells are flatter than control cells (Fig. 2f). We subsequently performed qRT-PCR analysis to document that this phenotypic transition parallels gene expression changes known to occur during EndMT, such as down-modulation of endothelial genes, like Vascular Endothelial-Cadherin (*VEC*; 74% of control cells) and Cluster of Differentiation 31 (*CD31*; 71% of control cells), induction of stem cell markers like *CD10* (28.6-fold upregulation) and *CD90* (2.1-fold upregulation)^37^, and induction of mesenchymal markers such as Fibroblast-Specific Protein 1 (*FSP1*; 20.3-fold upregulation), α-Smooth Muscle Actin (*αSMA*; 1.7-fold upregulation) and transcription factor *Slug*^18^,^38^,^39^ (34.7-fold upregulation; Fig. 2e). We further confirmed this characterisation at the protein level by immunofluorescence staining for *Slug* (Fig. 2d).

### MSX1 overexpression results in reduced cell-cell interactions consistent with EndMT

**Figure 3:**
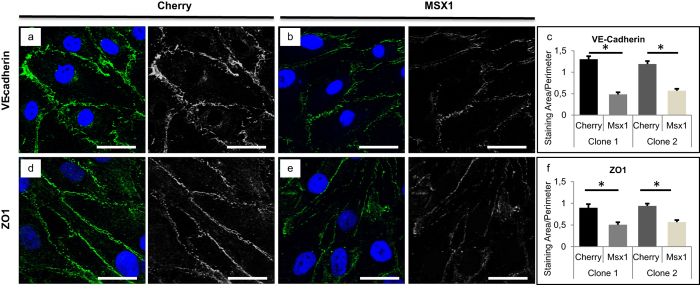
Disassembly of cell-cell contacts upon upregulation of MSX1 in a HUAEC monolayer. (**a**–**c**) Immunofluorescence staining on HUAECs transduced with Cherry- (**a**; control) or MSX1-encoding (**b**) lentivirus for the adherens junction marker VEC (green) and nuclear marker TO-PRO-3 (blue) and the corresponding quantification in (**c**). A reduced expression of VEC is observed in MSX1-overexpressing cells. Data in (**c**) are expressed as mean area (in μm^2^) per cell perimeter (in μm) ± s.e.m. of the total number of measured cells (*n* = 16 for all conditions; *P < 0.05 *versus* corresponding control by Student’s *t*-test). (**d**–**f**) Staining of Zonula Occludens-1 (ZO1; green) and nuclear marker TO-PRO-3 (blue). MSX1-overexpressing cells (**e**) show a reduction of ZO1 expression as compared to control cells (**d**). Panel (f) shows the corresponding quantification in which data are expressed as mean area of positive ZO1 signal (in μm^2^) per cell perimeter (in μm) ± s.e.m. of the total number of measured cells (*n* = 16 for all conditions; *P < 0.05 *versus* corresponding control by Student’s *t*-test). Scale bars: 25 μm.

In the early stages of EndMT, cell-cell contacts are disassembled resulting in a more motile, mesenchymal phenotype^40^. The loss of cell junctions is molecularly characterised by reduced expression of the adherens junction protein VEC^18^, and the diffusion of zonula occludens, namely ZO1^26^. We observe this reduction upon overexpression of MSX1 by immunostaining for VEC and ZO1 (Fig. 3a,b,d,e). Overexpression of MSX1 resulted in a 50% and 45% reduction of the area taken up by VEC and ZO1 staining at the cell perimeter, respectively, and this reduction was similar in both HUAEC clones (Fig. 3c,f). While reduced expression was most prominent in areas where cells were no longer in contact with neighbouring cells, there was also a reduction in expression in regions where cells were still in contact (Fig. 3b,e). The reduction in ZO1 protein expression was further confirmed by Western blot (Supplementary Fig. S1).

**Figure 4:**
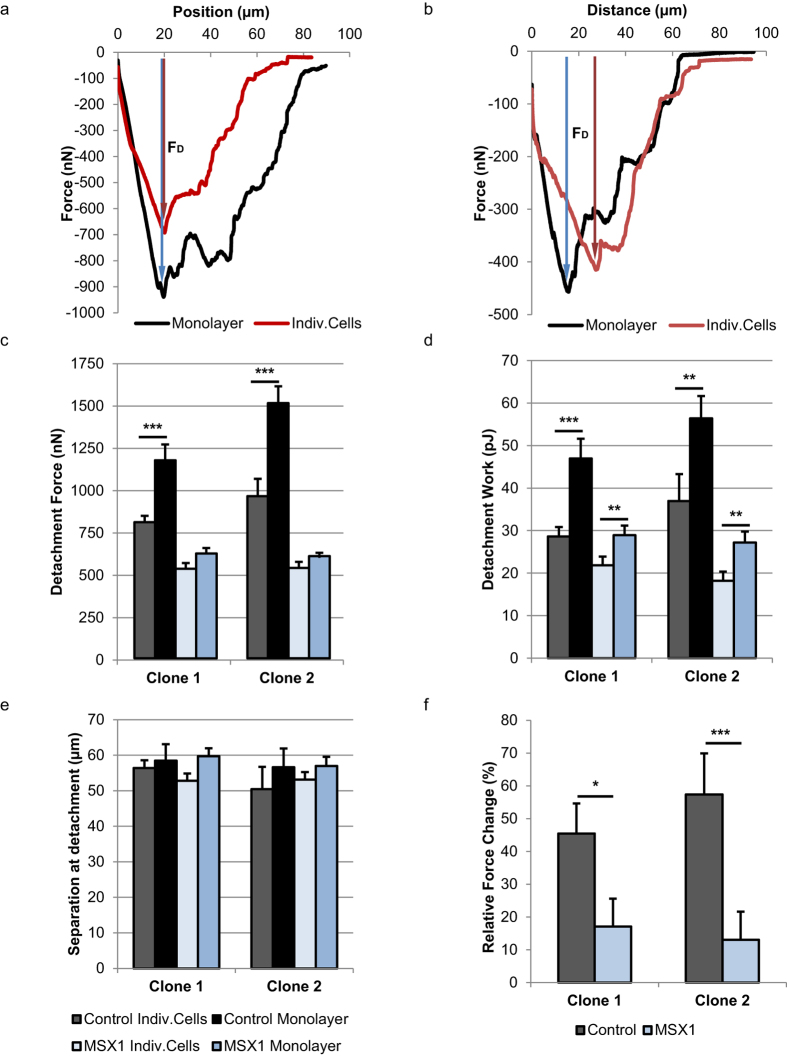
Decrease of cell-cell adhesion forces upon upregulation of MSX1 in a HUAEC monolayer. (**a**,**b**) Representative force curves registered during detachment of control cells (**a**) and MSX1-overexpressing cells (**b**). The red line refers to detachment of cells in individual state and the black line to cells in monolayer. Detachment force (F_D_), considered throughout this study as the adhesion force of a cell, is indicated by arrows and represents the maximum peak force recorded during retraction. (**c**) The average detachment force measured in control cells and MSX1-overexpressing cells in monolayer and individual cell states. (**d**) Average detachment work of control and MSX1-overexpressing cells. Detachment work is calculated by integrating the area demarcated by the force curve. (**e**) Separation at point of detachment for every cell condition. It refers to the separation between the cantilever tip and the maximum indentation point registered right before the last detachment event occurs. Same legend applies for (**c**–**e**). (**f**) Intercellular adhesion forces expressed as relative force change between cells in monolayer and individual cells. Cell-cell adhesion forces are reduced upon overexpression of MSX1. Error bars represent s.e.m. of the total amount of cells ‘*n*’ measured per condition, *n* = 30 in clone 1 and *n* = 43 in clone 2. *P < 0.05, **P < 0.03, ***P < 0.0007 by Student’s *t*-test for comparison of groups with unequal variances.

We have biomechanically characterised the cell-cell interactions using FluidFM technology, by analysing the detachment force curves of individual cells and cells in monolayer (Fig. 4a,b; Supplementary Videos SV3 and SV4; Supplementary Fig. S2). Both control and MSX1-overexpressing cells, either in monolayer and individual cell state, showed multiple detachment events visible in the retraction force curves as small force peaks. They indicated the progressive detachment of the mature cell adhesions from the substrate and rupture of the mature intercellular junctions. Concatenating rupture events until the complete detachment of the cell indicated that during the detachment process the cell is still active, despite the fact that cells could not be recovered afterwards. From these force curves we calculated the maximum detachment force (F_D_) of cells in the different conditions and used it as the main indicator of cell adhesion force (Fig. 4c). While control cells showed a very remarkable and significant increase in detachment of cells in monolayer compared to single cells, MSX1-overexpressing cells did not show any significant increase. Based on these values, we calculated the cell-cell adhesion forces as explained before (equation (1), Fig. 1a–d). The calculated intercellular forces were 365 nN for control cells and 90 nN for MSX1-overexpressing cells in clone 1; and 550 nN and 70 nN, respectively, in clone 2. According to equation (2), this corresponded to a relative force change of 45% in control cells and 17% in MSX1-overexpressing cells, in clone 1, and 57% and 13%, respectively, in clone 2 (Fig. 4f). By contrast, the separation at the point of detachment, which indicates to a certain extent the distance required for detachment, did not vary significantly (Fig. 4e). This parameter is strongly dependent on the cell size and stiffness, and is according to our results little influenced by the cell-cell contacts. The detachment work, being the area demarcated by the force curve, is also influenced by the size and stiffness of cells. Still, cell-cell interactions had a noticeable influence on this parameter, because the intercellular adhesion forces contribute to the transitory detachment forces and the widening of the force curve. As a result, a significant increase in detachment work between cells in monolayer and individual cells was visible in control and MSX1-overexpressing cells, although this increase was more moderate in the latter (Fig. 4d). Altogether, these data indicate that cell-cell adhesion strength decreases significantly during early stages of the EndMT process, which directly correlates with the disassembly of intercellular contacts which is known to occur during EndMT.


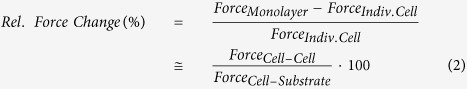


### MSX1 overexpression induces cytoskeletal and cell stiffness changes consistent with EndMT

**Figure 5:**
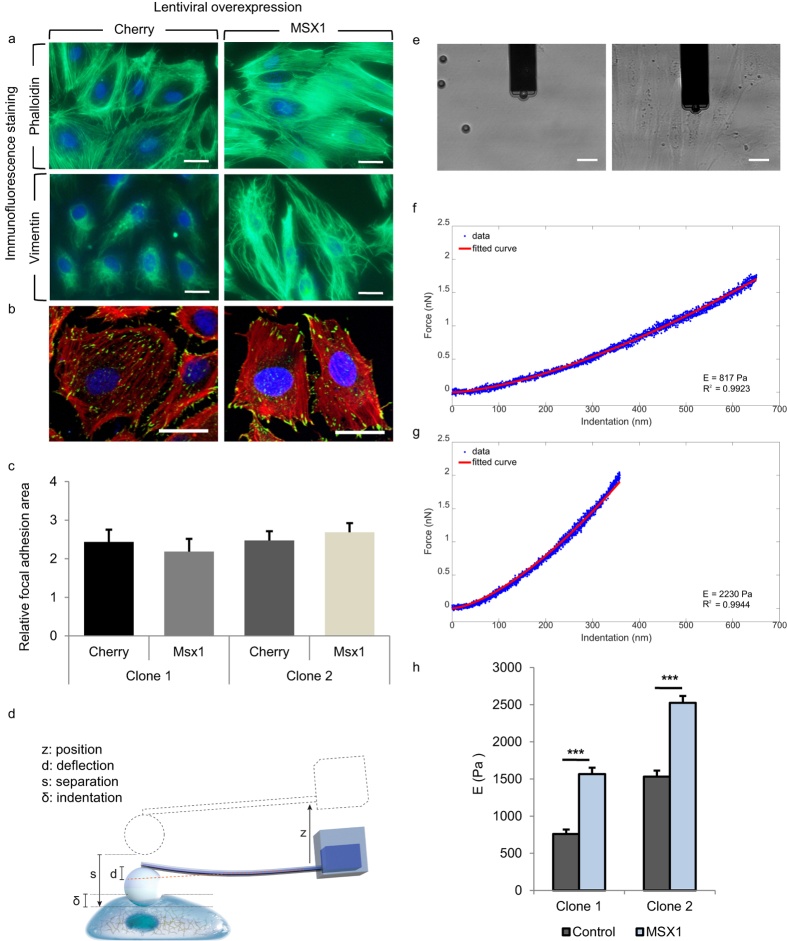
Reorganisation of the cytoskeleton and increased cellular stiffness upon upregulation of MSX1 in a HUAEC monolayer. (**a**) Immunofluorescence staining on HUAECs transduced with Cherry- (control) or MSX1-encoding lentivirus for nuclear marker Hoechst (blue) and phalloidin (green) in the actin cytoskeleton (top) and for vimentin (green) in the intermediate filaments (bottom). Scale bars: 25 μm. (**b**) Immunofluorescence staining on Cherry- (left) or MSX1-encoding lentivirus (right) for nuclear marker TO-PRO-3 (blue), cytoskeletal marker phalloidin (red) and focal adhesion marker vinculin (green). Scale bars: 25 μm. (**c**) Quantification of the fractional area of FAs in HUAECs from two clones of control and MSX1-overexpressing monolayers. Data are expressed as mean cumulative vinculin-positive area (in μm^2^) per cell area (in μm^2^) ± s.e.m. of the total number of measured cells (*n* = 16 for all conditions; *P* = not significant). (**d**) Diagram indicating the nomenclature for colloidal indentation. Z: vertical displacement of the engine; d: cantilever deflection; s: separation (s = z − d); δ: indentation, corresponding to the fraction of the separation after the contact point with the cell. (**e**) 4 μm opening cantilever and 10 μm beads in a petri dish with culture medium; the cantilever has a bead immobilised at the tip (left). Cantilever and bead indenting on a monolayer of MSX1-overexpressing cells (right). (**f**,**g**) Representative examples of the fitting of *Force-Indentation* curves with Hertz model for control (**f**) and MSX1-overexpressing cells **(g**). Deeper indentations in the cell and smoother slope indicate lower stiffness. Insets specify the calculated apparent Young’s modulus and goodness of fit. (**h**) Cell stiffness measured by colloidal indentation. Bars represent the mean value of the collected data set and error bars indicate s.e.m. The statistical analysis was performed on the logarithmically transformed data due to a non-Gaussian distribution of the original data set (Supplementary Fig. S4). To facilitate the reading, the original values are displayed in the diagram instead of the logarithmically transformed data. Clone 1: ***P < 10^−16^, Student’s *t*-test, *n* = 74, 105 cells for Cherry-(control) and MSX1-overexpression, respectively; clone 2: ***P < 10^−13^, Student’s *t*-test for comparison of groups with unequal variances, *n* = 119, 155 cells for Cherry-(control) and MSX1-overexpression, respectively.

Lastly, we investigated if MSX1 overexpression resulted in cytoskeletal rearrangements known to occur during EndMT^35^. We detected cytoskeletal changes upon MSX1 overexpression in HUAECs *in vitro* by staining for phalloidin, which binds to the filaments and bundles of the actin cytoskeleton. We found that MSX1-overexpressing cells had increased formation of stress fibres where F-actin filaments were assembled parallel to the long axis of the cells. By contrast, F-actin filaments in control cells were more localised in the cell periphery near cell-cell junctions (Fig. 5a, top). We also found a change in the intermediate filament composition through an increase in vimentin content. This agrees with previous data described for EMT processes where intermediate cytokeratin filaments are replaced by vimentin containing filaments^26^ (Fig. 5a, bottom). In addition to these changes in intermediate filament composition and cytoskeleton organisation, we also looked for a potential change in the FA configuration, as this would be expected to occur upon acquisition of a more motile phenotype during EndMT^41^. We found that while the total area of the cell bottom taken up by FA was not altered by MSX1-overexpression (Fig. 5c), this intervention caused a redistribution of FA towards the cell edges (Fig. 5b). Furthermore, we biomechanically characterised the cells by SCFS through the apparent Young’s modulus as a characteristic parameter of cell stiffness. The measurements were performed by immobilising a microbead at the tip of the FluidFM cantilever and performing small indentations on the nuclear region of the cells (Fig. 5d,e). Indentations were performed until the set point force of 2 nN was reached. Force curves from the approach regime were extracted and processed (Supplementary Fig. S3 and Supplementary Note 1). The apparent Young’s modulus was then calculated by fitting the *Force (nN*) - *Indentation (nm*) curve with the modified Hertz model for spherical indenters^42^ (equation (3), Fig. 5f,g), where F is the force, *E*_*y*_ the Young’s Modulus, *R* the radius of the spherical indenter, *δ* the indentation and *ν* the Poisson’s ratio, set as 0.5 for this study^43^.


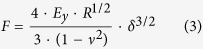


By calculating the apparent Young’s Modulus, we found that MSX1-overexpressing cells were significantly stiffer than control cells (Fig. 5h), its value being around double than that of control cells (from 760 Pa to 1,530 Pa in clone 1 and from 1,565 Pa to 2,523 Pa in clone 2). Our results show that, in the early stages of the EndMT process, the elastic properties of HUAECs had already changed resulting in increased stiffness. These changes agree with known cytoskeletal reorganisation that enables dynamic cell elongation and directional motility^40^. Moreover, these changes correlate with the general relationship between increased cytoskeleton organisation and higher Young’s Modulus previously described for other biological contexts^44^. (See Supplementary Fig. S4 for histograms used in this study).

## Discussion

In this study, and as already introduced by other groups, we show that the FluidFM technology overcomes the limits of conventional AFM for cell immobilisation and, therefore, strongly adherent cells can be detached from the substrate and their adhesion forces can be measured^8^,^9^. Moreover, cells in monolayer can be completely detached making it possible to assess intercellular adhesion forces of well-established mature junctions. By this means, we have introduced a new methodology to measure the cell-cell adhesion forces, consisting of the difference between the adhesion forces exerted by cells in monolayer and that of individual cells. While this approach does not take into consideration the interplay between cadherins and integrins^29^ nor the area reduction of cells in monolayer compared to individual cells, our method provides a solid approximation to the overall intercellular adhesion force of a cell within a monolayer, which opens new opportunities for the biomechanical characterisation of cells within tissue-like constructs. Noteworthy, unlike in a previous study^45^, we did not observe a decrease in the number of FAs upon reaching confluence, suggesting that under our working conditions a difference in FA-based contact with the cell substrate was not a major confounding factor in our measurement of cell-cell adhesion forces.

In the biological context, we describe for the first time the biomechanical changes that occur during early stages of EndMT. This study was performed on primary arterial endothelial monolayers upon overexpression of transcription factor MSX1, and biomechanical changes were correlated with the more routinely performed biomolecular analyses. MSX1-overexpression induced a phenotypic transition in these cells corresponding to EndMT, altering cell morphology towards a spindle-like shape and reducing cell height. Biochemical analyses confirmed this transition by showing a reduction in endothelial markers and an increase in stem cell and mesenchymal markers at the mRNA and protein levels. This novel methodology for biomechanical characterisation allowed, for the first time, the measurement of the absolute intercellular adhesion forces of cells in a monolayer, revealing a significant reduction of cell-cell forces in the early stages of EndMT. These results, together with the decrease of endothelial cadherins and the diffusion of zonula occludens observed by immunostaining and Western blot, verified the disassembly of cell-cell adhesive structures that typically occur during EMT^46^,^47^. Additionally, we measured the apparent Young’s Modulus during the early stages of EndMT, induced by MSX1 overexpression and it revealed a significant increase in the stiffness of HUAECs as a consequence of the transition. This was associated with a reorganisation of the cytoskeleton shown by an accumulation of actin bundles, an increase in vimentin content and a redistribution of FAs towards the cell edges. While, to the best of our knowledge, the latter observation has not been reported before in the context of EndMT, it is known that FA formation at the edges of the cell is important to coordinate EC migration^41^ and that the acquisition of a migratory phenotype is part of the EndMT process^48^. Alterations in cell stiffness have been reported both for EMT and EndMT that show different tendencies, some indicating an increase and others a decrease^49^,^50^,^51^,^52^. Although we presume cell type and cell malignancy are contributing factors to this differential effect, a deeper understanding of these processes is still required. Our results suggest that overexpression of MSX1 is sufficient to induce EndMT *in vitro* and that its ability to induce it is not limited to the endocardium, where it was previously shown to contribute to atrioventricular valve formation^33^,^34^.

In conclusion, we introduce a novel methodology to measure the intercellular adhesion forces that cells exert in confluent monolayer by using fluidic probe force microscopy. This method enables the measurement of high adhesion forces of several thousand nanonewton per cell, so that intercellular adhesion forces can be assessed by subtracting cell-substrate adhesion forces from the forces measured when removing a cell from a monolayer. We apply this method to a new *in vitro* model of EndMT as a relevant multicellular context and biological test case. There, we quantify for the first time the involved cell-cell adhesion forces and stiffness of the cells in the early phase of EndMT in monolayer, as a representative model of the process that occurs *in vivo*. These biomechanical studies are complemented with conventional biochemical and morphological analysis. Our unprecedented quantitative findings present a significant and important technical advancement as a new tool in the field of mechanobiology for direct measurement in a physiological setting. They establish a strong foundation for future correlative quantitative studies of a variety of biologically relevant questions in development, fibrosis, physiology and the origin and progression of diseases such as cancer, as well as in the study of cell-biomaterial interaction. We envision that technological advancement will make it possible to assess intercellular forces also at the tissue level.

## Methods

### EC isolation

This study complies with the Helsinki Declaration with ethical approval from the Ethical Committee of the University Hospital Leuven (UZ Leuven; approval number: B32220084019). Written informed consent was obtained from all umbilical cord donors included in this study. First, vessels were flushed with PBS to rinse out the blood and subsequently filled with 0.2% collagenase type I (Gibco) dissolved in 0.9% NaCl with 2 mmol/L CaCl_2_, and then incubated at 37 °C for 12 minutes to detach the ECs. The collagenase suspension containing ECs was collected by flushing the vessels with PBS with 1% FBS, filtered through a 40 μm cell strainer and spun down at 600 g for 7 minutes. Next, cells were plated on flasks pre-coated with 0.1% gelatin (Sigma-Aldrich) using EGM 2MV culture medium supplemented with the CC 4147 Bulletkit (Lonza) and 1% penicillin/streptomycin (Life Technologies).

### Lentiviral production and transduction

The MSX1-encoding lentiviral plasmid was made by cloning its open reading frame from a cDNA containing plasmid (Thermo Scientific) behind the cytomegalovirus (CMV) promoter in a pRRL2 CMV PGK Cherry backbone. For virus production, human embryonic kidney (HEK) 293 cells (5 × 10^6 ^cells/10 cm dish) were plated on Day 0. On Day 1 they were transduced with pRRL2 CMV MSX1 PGK Cherry or Cherry control plasmid, and the viral packaging plasmids (psPax2 and PMD2G) using Fugene transfection reagent (Roche). On Day 2 the medium was replaced and the viral particle-containing supernatant was collected 36 hours later. Once the lentiviral constructs were generated, HUAECs (25,000 cells per well of a 24 well plate) were plated on Day 0 in normal growth medium. For transduction, cells were incubated with viral supernatant on Day 1; then, transduced cells were rinsed on Day 2 and Day 5. Finally, cells were harvested by trypsinisation to be used in the biomechanical experiments and for immunofluorescence staining or Western blot after reseeding of the cells.

### Immunofluorescence staining

For immunofluorescence imaging, 6,000 (for the sparse condition) or 40,000–60,000 (for the confluent monolayer condition; density range is dependent on the type of culture vessel) lentivirally transduced HUAECs were seeded in gelatin-coated chambers of a 4-well Millicel EZ slide from Millipore (PEZGS0416) or in gelatin-coated 24-wells, washed with Dulbecco’s PBS (D-PBS) and fixed for 5 minutes with 1% PFA or 10 minutes with 4% PFA according to the staining protocol. Next, cells were washed with TBS/glycine (composition of 10 X stock solution: 38 g NaCl, 9.38 g Na_2_HPO_4_, 2.07 g NaH_2_PO_4_ and 37.5 g glycine), and incubated with blocking solution (2% BSA in TBS) for one hour. Cells were incubated overnight at room temperature with primary antibodies against MSX1 from R&D Systems (AF5045, 1:20) in blocking solution. The following day, the cells were washed and incubated with Alexa-488 conjugated secondary antibody (1:500) for 1 hour. Then, they were washed, incubated for 5 minutes with Hoechst 33253 solution from Sigma-Aldrich (94403, 1:500) and washed again. Rinsing steps following incubation with primary antibodies were performed with IF wash buffer (composition of 10 x stock solution: 38 g NaCl, 9.38 g Na_2_HPO_4_, 2.07 g NaH_2_PO_4_, 2.5 g NaN_3_, 5.0 g BSA, 10 mL triton X 100 and 2.5 mL Tween 20).

Alternatively, after the PFA fixation cells were washed with D-PBS. Only for SLUG staining cells were permeabilised with 0.5% triton in D-PBS for 5 minutes, washed again with D-PBS and incubated with blocking solution (2% BSA in D-PBS) for one hour. Next, cells were incubated overnight at 4 °C with primary antibodies against SLUG from Cell Signalling (C19G7, 1:50), VE-cadherin from Abcam (ab7047, 1:50), ZO1 from ThermoFisher Scientific (40–2200, 1:25), vimentin from Sigma (V5255, 1:100), vinculin from Sigma (V5135, 1:100) or Alexa Fluor488 or Rhodamine-conjugated phalloidin from ThermoFisher Scientific (A12379 and R415, respectively, 1:100) in blocking solution. Then, they were washed and incubated for 1 hour with Alexa-488 conjugated secondary antibody from Life Technologies (1:500) or, alternatively for staining of SLUG with biotin conjugated secondary antibody from Santa Cruz (1:300). For the latter, signals were amplified with Fluorescein tyramide based amplification systems (Perkin Elmer, NEL701A). After washing, cells were incubated for 5 minutes with TO-PRO-3 from ThermoFisher Scientific (T3605, 1:1,000; for confocal images) or Hoechst 33253 solution from Sigma Aldrich (94403, 1:500; for images on a regular fluorescence microscope) and washed again. After staining, cells were mounted with ProLong Gold Antifade reagent from ThermoFisher Scientific (P36934). Pictures were taken on a Zeiss Axiovert 200 M fluorescence microscope (Carl Zeiss) or an LSM 700 confocal microscope (Zeiss 200).

### RNA isolation and qRT-PCR

For gene expression analysis, 200,000 lentivirally transduced HUAECs were seeded in a 6 well plate, grown until confluency and then lysed. Total RNA was extracted and isolated using a TRIzol reagent-based protocol (Invitrogen, 15596-018). RNA was isolated and next reverse transcribed using Superscript III Reverse Transcriptase (Invitrogen). For expression analysis by qRT-PCR, cDNA was amplified using the primer sequences listed in Supplementary Table 1 during 40 cycles on a Step One Plus RTPCR system (Applied Biosystems) and detected by intercalation of the fluorescent SYBR Green I dye (Applied Biosystems) in the dsDNA. Each measurement was performed in triplicate and mRNA levels were normalised against *GAPDH* as housekeeping gene.

### Western Blot

Lentivirally transduced HUAECs were seeded at a density of 200,000 cells in a gelatin-coated well of a 6-well plate and grown until confluency. Cells were lysed in Laemlli buffer supplemented with Protease and Phosphatase inhibitor cocktail from Sigma (P8340, 1:200 and P2850, 1:100, respectively). The BCA protein assay kit (Thermo Scientific, #23225) was used to determine the protein concentration. Subsequently, samples were mixed with reducing agent (Life Technologies, NP009) and LDS Sample Buffer (Life Technologies, NP007), loaded on a NuPAGE Novex 4–12% Bis-Tris Protein Gel from ThermoFisher Scientific (NP0321PK2) and run for 90 minutes at 130 V. Subsequently, proteins were blotted on a nitrocellulose membrane during 90 minutes at 90 V. Membranes were washed with TBS-Tween and blocked with 5% milk in TBS-Tween for 1 hour at RT. Primary antibodies were incubated overnight at 4 °C. The next day, membranes were washed and incubated with Horse Radish Peroxidase-conjugated secondary antibodies from Santa Cruz diluted in 0.5% milk in TBS-Tween. The signal was revealed using the Pierce ECL Western Blotting Substrate from ThermoFischer Scientific (32106) and pictures of the membranes were taken on a Bio-Rad ChemiDoc XRS^+^ molecular imager with Image Lab software (Bio-Rad Laboratories). The following primary antibodies were used: β-Tubulin from Cell Signalling (D2N5G, 1:1,000) as loading control, MSX1 from R&D systems (AF5045, 1:200) and ZO1 from ThermoFisher Scientific (40–2200, 1:1,000). Image Lab 4.0 software was used to quantify band intensity and bands were normalised relative to the loading control.

### Microscopy and morphometric analysis

Images were recorded at room temperature on a Zeiss AxioImager Z1 microscope with EC Plan Neofluar objective lenses at 20x (NA 0.5) and 40x (NA 0.75) magnification and on a Zeiss Axiovert 200 M fluorescence microscope with LD Plan-Neofluar objective lenses at 20x (NA 0.4) and 40x (NA 0.6) magnification, both equipped with a Zeiss AxioCam MRc5 camera (Carl Zeiss). The acquisition was performed with Axiovision 4.8 software (Carl Zeiss). To determine cell surface expression of adherens junction molecules, the fraction of VEC or ZO1 on the cell perimeter was measured on 16 randomly selected cells from 4 randomly selected areas per condition for each of the two clones, using open software for image processing ImageJ (http://imagej.nih.gov/ij/). Data were expressed as the area positive for VEC or ZO1 (in μm^2^) normalised to the cell perimeter (in μm). To determine the number of FAs or the cumulative area taken up by FAs, the number of vinculin-positive FAs or the area with vinculin signal, respectively, were measured in 10 (for the analysis of the effect of seeding density) or 16 (for the analysis of the effect of Msx1-overexpression in monolayers) randomly picked cells from 4 randomly selected areas, using ImageJ software. Data were expressed as the number of vinculin-positive spots or the cumulative vinculin-positive area (in μm^2^) per cell area (in μm^2^; determined based on the phalloidin cytoskeletal staining).

For the calculation of the geometrical parameters of cells, brightfield images of EC monolayers were taken where the contour of cells was marked out using ImageJ. Area, perimeter and axes of the cells were automatically calculated as well as their aspect ratio, circularity and roundness.

### Single Cell Force Spectroscopy (SCFS)

#### Device

All measurements regarding cell adhesion forces, cell elasticity and cell height were performed by Single Cell Force Spectroscopy (SCFS) using a Flex FPM (Nanosurf GmbH, Germany) system which combines AFM with FluidFM technology (Cytosurge AG, Switzerland). The system was mounted on an Axio Observer Z1 inverted microscope (Carl Zeiss, Germany) and a piezoelectric stage of 100 μm retraction range. Rectangular, 200 μm long and 36 μm wide hollow cantilevers of different opening and nominal stiffness were selected according to the type of measurements. The spring constant was calibrated based on the thermal noise method^53^ executed in Nanosurf software-implemented tools. The deflection sensitivity of the cantilever was calibrated before every experiment by a Cytosurge software built-in procedure. Cantilevers were loaded with ultrapure water in their reservoir.

#### Cell seeding for SCFS

L929 cells were seeded on cover glasses of 15 mm in diameter at densities of 50,000 and 100,000 cells/cm^2^. In the case of HUAECs cover glasses were first incubated at 37 °C with gelatin at least one hour and then seeded at densities between 20,000 to 50,000 cells/cm^2^. Glasses were transferred to individual petri dishes prior to the measurements, which were performed in culture medium between days 1 and 3 after cell seeding. These cell density ranges were set based on the size and proliferation of the cells in order to have samples with individual cells and monolayers for the three days of measurements. Each sample was used no longer than two hours while measuring.

#### Adhesion force of cells

The adhesion force of cells was assessed using Micropipette cantilevers with an aperture of 8 μm in diameter and a nominal spring constant of 2 N/m. Prior to every measurement, the cantilever was set at a distance of 45 μm away from the sample. A cell was selected and approached at 5 μm/s until a set point of 70 nN was reached, or 50 nN for L929 cells. The approach was followed by a pause of 3 s and a suction pressure of 800 mbar to ensure sealing of the cell to the cantilever, while the position of the stage was maintained constant. Then, while maintaining the suction pressure, the cantilever was retracted 95 μm at a speed between 2 and 10 μm/s. The retraction speed was adjusted for each cell type based on handling criteria, setting it at 5 μm/s for L929 cells, 10 μm/s for clone 1 and 2 μm/s for clone 2, for both control and MSX1-overexpressing cells. Data were collected at a frequency of 512 Hz. After every cell, the cantilever was cleaned 5 minutes in 4% sodium hypochlorite solution (NaClO) and rinsed 3 times in ultrapure water. The retraction curve was extracted on SPIP 6.2.0 software (Image Metrology, Denmark) and displayed as Force (nN) Position (nm), Position meaning the displacement of the stage for the retraction of the cantilever. The force F [N] is proportional to the deflection of the cantilever, which is provided by the photodetector in the form of electric signal, and fulfils Hook’s Law F = k∙x = k∙d∙ν, where k is the spring constant [N/m] and x the deflection of the cantilever [m] after multiplying the deflection signal d [V] by the deflection sensitivity v [m/V]. The maximum deflection of the cantilever during retraction provides the so called maximum detachment force (FD) of a cell^6^, which is the parameter commonly used as representative for adhesion force. From the same force curves, the detachment work and separation at detachment were also calculated. The detachment work is the area demarcated by the force curve; the separation at detachment is considered as the separation of the cantilever as registered right before the last detachment event. The three parameters were automatically calculated on SPIP. Between 30 and 43 cells per condition and clone were collected.

#### Modulus of elasticity of the cell

The Apparent Young’s Modulus of cells was calculated from the approach curve of the spectroscopy cycle. Micropipette cantilevers with nominal spring constant of 0.2 N/m and an aperture of 4 μm in diameter were used. As an indenter, a polyethylene glycol coated polystyrene bead of 10 μm in diameter (Micromer #01-54-104, Micromod Partikeltechnologie GmbH, Germany) was immobilised at the aperture of the cantilever as follows^54^: beads at a density of 5 μg/mL were added into a petri dish containing culture medium. A bead was grabbed by applying a suction pressure of 700 mbar and the deflection sensitivity was calibrated. Next, the petri dish was exchanged by the one containing the cover glasses with the cells (Fig. 5e). Indentations were performed on the nucleus of the cells in monolayer starting the cantilever at a distance of 5 μm. The approach was done with the internal AFM headset engine at 500 nm/s, until a set point of 2 nN was reached, with a data acquisition frequency of 2,000 Hz. A pause of 2 s and a retraction distance of 5 μm followed every indentation. During the experiment the bead was retained at the tip with a suction pressure of 300 mbar.

#### Data processing

For the calculation of the apparent Young’s Modulus, the approach curve of the indentation experiment was processed with a custom programme written in Matlab 2015b (Mathworks, USA) and fitted according to the Hertz model for spherical indenters^42^,^55^ (equation (3)). First, the approach curve was extracted and displayed in the form of Force (nN) Separation (nm), Separation meaning the subtraction of the cantilever displacement and the cantilever deflection (Fig. 5d). Then, the baseline was corrected and the contact point in the vertical direction of the indenter onto the cell was calculated by the method of Contact Point Extrapolation (CPE)^56^,^57^, which is based on a linear relationship between Force and Separation (see Supplementary Note 1). This linear relationship was plotted and the CPE method applied. The calculated contact point of every curve was visually verified and corrected, if needed. Based on the calculated contact point, the approach curve was then represented as a function of Force (nN) Indentation (nm); Indentation being the Separation starting at the contact point on the surface of the cell and up to the depth at which the given set point was reached. Finally, this Force Indentation curve was fitted with the Hertz model for spherical indenters (equation (3)) and from there the apparent Young’s Modulus was extracted. The curve fitting range was set between 10 nm and 370 nm, to ensure indentations within ~15% of cell height. Curves with a goodness of fit (R2) below 0.95 were discarded.

#### Cell height

For the calculation of cell height colloidal indentations were performed as for the calculation of the modulus of elasticity, and same cantilevers, beads and spectroscopy parameters were used. Starting at a height of 5 μm from the surface of the cell, an indentation at 500 nm/s was performed on a spot of glass surface found within the monolayer, until a set point of 2 nN was reached. From the approach curve of this indentation the contact point in the vertical direction where the indenter reaches the glass surface was calculated, which indicates the separation between these two elements. Next, without moving the stage and starting at the very same cantilever position, an indentation on the nucleus of the cell next to it was performed at equal speed and set point as before. From the approach curve of this second indentation, the contact point at which the indenter reaches the cell was calculated, which indicates the separation between them. The difference between the separation from the indenter to the nucleus of the cell and to the glass slide shows the height of the cell at the nucleus region^58^. The contact points for the calculation of the cell height were obtained through the same programme written in MATLAB used for the calculation of the modulus of elasticity.

### Statistics

In the biomolecular analyses, data are represented as mean ± s.e.m. and *n* indicates the number of biologically independent experiments or cells. The fold inductions determined qRT-PCR were statistically analysed by means of a one sample Student’s *t*-test with null-hypothesis 1. Prism software was used for statistical analysis and a *P*-value lower than 0.05 was considered significant. In the SCFS experiments performed with FluidFM for adhesion forces, cell elasticity and cell height, the normality of the data groups under comparison was first evaluated by Shapiro Wilk test. Outliers were identified according to Grubbs’ test with a significance level of 0.05 and accordingly discarded if any. Parametric analyses were performed on the untransformed data in case of normally distributed data sets. Equality of variances was first evaluated based on Levene’s test. Subsequently, Student’s *t*-test was performed between groups with equal variances and, in the case of unequal variances, comparison of groups was performed by Welch’s *t* test, also known as Student’s *t*-test for unequal variances. A *P*-value lower than 0.05 was considered significant. In the case of cell stiffness, the calculated data in the form of apparent Young’s Modulus did not follow a Gaussian distribution according to the Shapiro Wilk test. Thus, data were logarithmically transformed to adopt a normal distribution, corroborated then by the same test. Next, a Student’s *t*-test was performed on the logarithmically transformed data, although the corresponding non transformed Young’s Modulus value of the compared means was displayed for a better comprehension by the reader. IBM SPSS Statistics software and Excel were used for the statistical analysis.

## Additional Information

**How to cite this article:** Sancho, A. *et al*. A new strategy to measure intercellular adhesion forces in mature cell-cell contacts. *Sci. Rep.* **7**, 46152; doi: 10.1038/srep46152 (2017).

**Publisher's note:** Springer Nature remains neutral with regard to jurisdictional claims in published maps and institutional affiliations.

## Supplementary Material

Supplementary Video 1

Supplementary Video 2

Supplementary Video 3

Supplementary Video 4

Supplementary Information

## Figures and Tables

**Table 1:** Geometrical characteristics of HUAECs.

		Area (μm^2^)	Perim. (μm)	Major Ax. (μm)	Minor Ax. (μm)	Aspect Ratio	Circular.	Round.
Clone 1	Control	6372 ± 275	321 ± 7	104 ± 2	75 ± 2	1.40 ± 0.02	0.75 ± 0.01	0.73 ± 0.01
	MSX1	4405 ± 114	380 ± 6	155 ± 2	36 ± 1	4.46 ± 0.11	0.39 ± 0.01	0.24 ± 0.01
Clone 2	Control	6187 ± 273	309 ± 7	109 ± 3	71 ± 2	1.55 ± 0.04	0.80 ± 0.01	0.66 ± 0.02
	MSX1	2077 ± 86	298 ± 7	123 ± 3	21 ± 1	6.02 ± 0.21	0.30 ± 0.01	0.18 ± 0.01

Cells from two independent clones were transduced with Cherry- (control) or MSX1-encoding lentiviruses (MSX1). *Aspect ratio* is the coefficient between the major and the minor axes, *circularity:*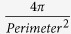, and *roundness:* 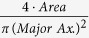. MSX1-overexpressing cells have a higher aspect ratio, and lower circularity and roundness than control cells. Values are displayed as mean ± s.e.m. of the total amount of cells ‘*n*’ measured; *n* = 100 for control and MSX1 in clone 1; *n* = 54, 71 in clone 2 for control and MSX1, respectively.
