# Supplementary material for: A new strategy to measure intercellular adhesion forces in mature cell-cell contacts
Source: Sci Rep. 2017 Apr 10;7:46152. doi: 10.1038/srep46152 (PMC5385528; doi:10.1038/srep46152)
Supplement: Supplementary Information [file srep46152-s5.pdf]

SUPPLEMENTARY INFORMATION

A new strategy to measure intercellular adhesion forces in mature cell-cell contacts

Ana Sancho[1], Ine Vandersmissen[2], Sander Craps[2], Aernout Luttun[2,*], Jürgen Groll[1,*]

[1] Department of Functional Materials in Medicine and Dentistry and Bavarian Polymer Institute (BPI), University of Würzburg, 97070 Würzburg, Germany
[2] Department of Cardiovascular Sciences, Center for Molecular and Vascular Biology, KU Leuven, 3000 Leuven, Belgium
[*] Corresponding authors

Video SV1: Detachment of a HUAEC from a monolayer. 8 µm aperture cantilever approaches a HUAEC and detaches it from a monolayer as explained in Video 1.

Video SV2: Detachment of a L929 fibroblast from a monolayer. 8 µm aperture cantilever approaches an L929 cell in a monolayer; then, it applies suction pressure, immobilises the cell at the tip and detaches it entirely during retraction. The gap left behind in the monolayer by the cell is visible in the video when the cantilever returns to its original position.

Video SV3: Detachment of a Cherry-encoding control endothelial cell from a monolayer. 8 µm aperture cantilever approaches a Cherry-encoding control HUAEC and detaches it from a monolayer as explained in Video 1.

Video SV4: Detachment of an MSX1-overexpressing endothelial cell from a monolayer. 8 µm aperture cantilever approaches an MSX1-overexpressing HUAEC and detaches it from a monolayer as explained in Video 1.

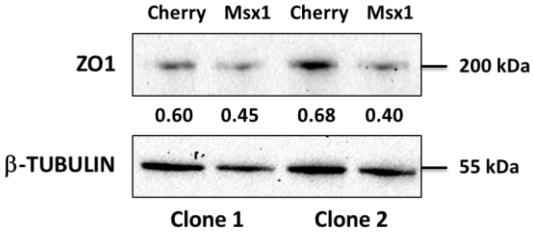

Figure S1: Western blot and densitometric quantification (normalised for β-tubulin as loading control) showing ZO1 protein expression in 2 HUAEC clones overexpressing MSX1 (lanes 2 and 4) and the corresponding controls (lanes 1 and 3) revealing a 25% and 40% down-regulation of ZO1 protein levels upon MSX1 overexpression.

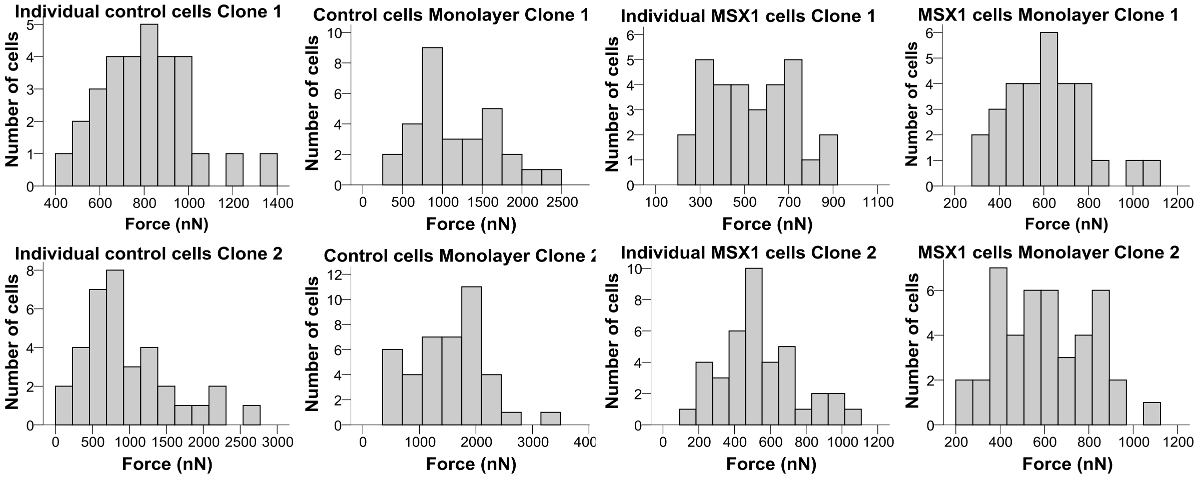

Figure S2: Histograms of the adhesion forces of individual cells and cells in monolayer, measured by detachment of cells using AFM with FluidFM® add-on technology.

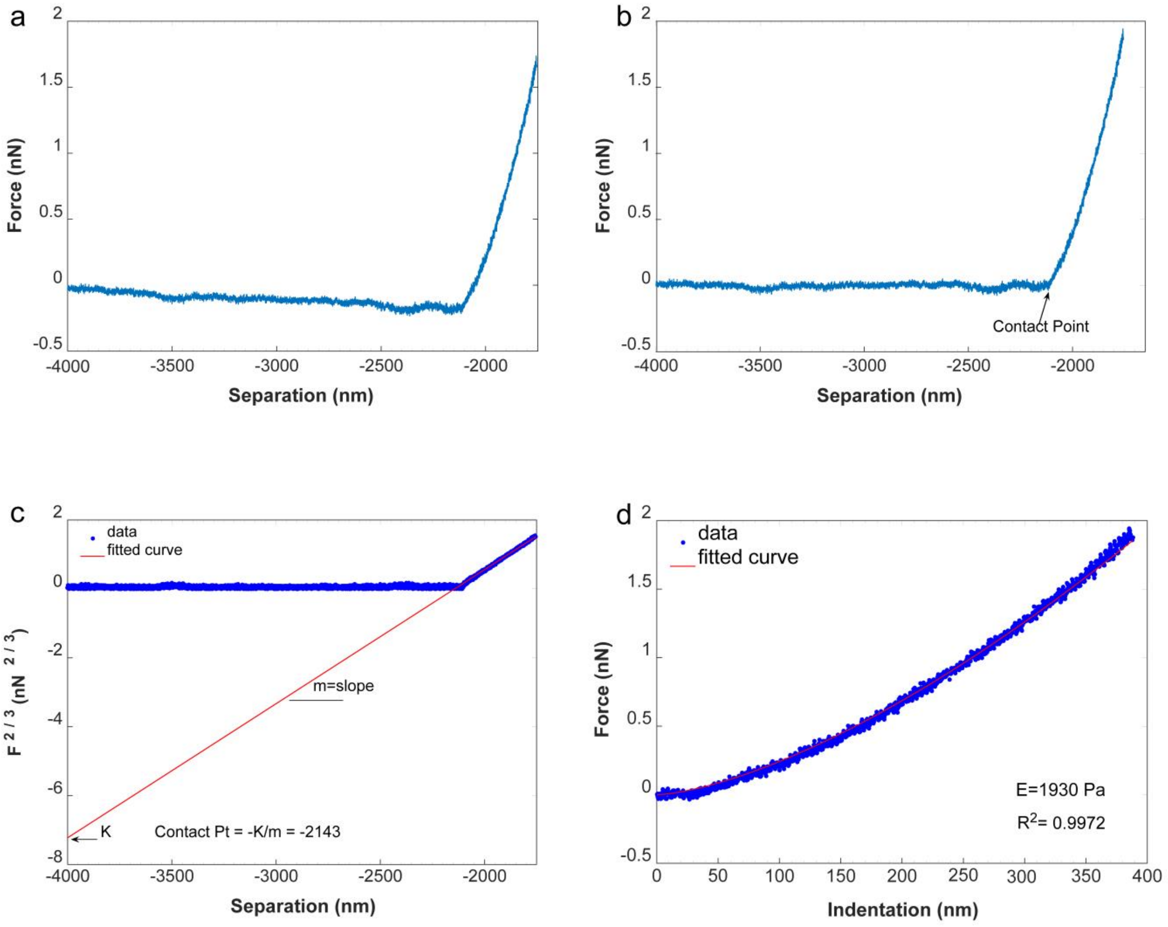

Figure S3: Representative sequence of the data analysis process performed in MATLAB for the calculation of the apparent Young's Modulus. The approach curve of the indentation cycle is taken and the cantilever deflection signal converted into force by multiplying it by the spring constant and the deflection sensitivity. (**a**) Based on the position

and deflection of the cantilever, data are expressed in Force/Separation mode. (**b**) Force/Separation curve after baseline correction. (**c**) Calculation of the contact point by extrapolation. (**d**) Force/Indentation curve obtained after the calculation of the contact point; this curve is fitted with a modified Hertz model for spherical indenters. With the curve fitting the apparent Young's Modulus can be directly calculated.

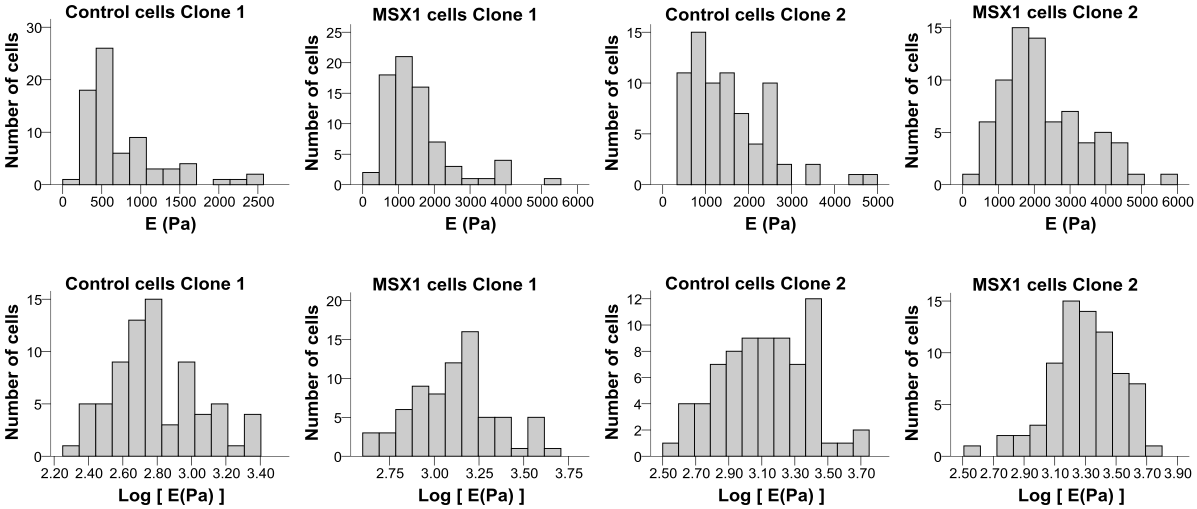

Figure S4: Histograms of the Modulus of Elasticity (above) measured by colloidal indentation on cells in monolayer using AFM with FluidFM® add-on technology; and the logarithmically transformed histograms (below) used for parametric statistical analysis.

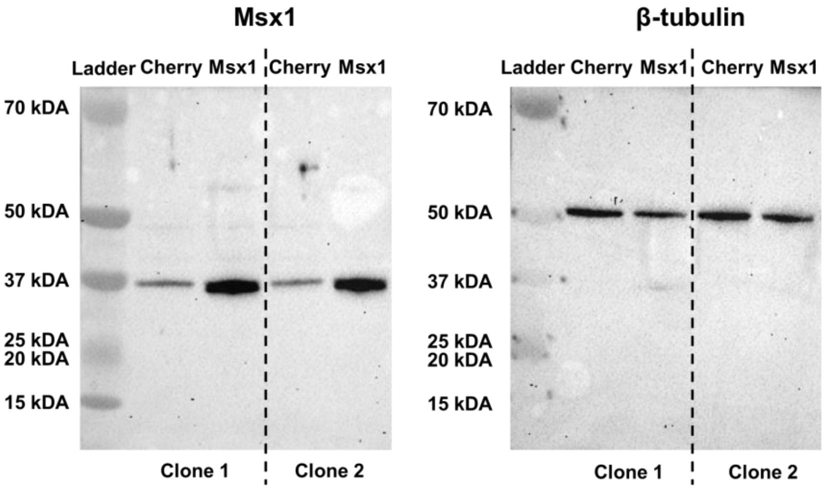

Figure S5: full-length Western blot showing Msx1 protein overexpression and β-tubulin as loading control.

Table 1. List of primers for qRT-PCR.

Gene	Forward primer (5'-3')	Reverse primer (5'-3')
αSMA	*TGGTGTGTGACAATGGCTCT*	*CTTTTCCATGTCGTCCCAGT*
CD10	*AATGTCATTCCCGAGACCAG*	*AGC TGT CCA AGA AGC ACCAT*
CD31	*TCTGCACTG CAGGTATTGACAA*	*CTGATCGATTCGCAACGGA*
CD90	*GACCCGTGAGACAAAGAAGC*	*GCCCTCACACTTGACCAGTT*
FSP1	*CAAGTACTCGGGCAAAGAGG*	*CTTCCTGGGCTGCTTATCTG*
MSX1	*AGTTCTCCAGCTCGCTCAGC*	*GGAACCATATCTTCACCTGCGT*
SLUG	*CATGCCATTGAAGCTGAAAA*	*GCAGTGAGGGCAAGAAAAAG*
VEC	*GTTCACGCATCGGTTGTTC*	*TCTGCATCCACTGCTGTCA*

Note 1:

Contact Point Extrapolation (CPE):

CPE is a method that permits to mathematically calculate the point of contact between the indenter and the surface of the sample based on the indentation data expressed in the form of Force-Separation. The power function that relates the force and the indentation in the Hertz model for spherical indenters is transformed into a linear function, as follows:

$$F = \frac{4E_y R^{1/2}}{3(1-\nu^2)}\delta^{3/2} \quad \rightarrow \quad F^{2/3} = \left[\frac{4E_y R^{1/2}}{3(1-\nu^2)}\right]^{2/3} \cdot \delta$$

where F is the force, E_y the Young's Modulus, R the radius of the spherical indenter, δ the indentation and ν is Poisson's ratio. Then, the indentation is expressed as the subtraction between the cantilever position (z) and the deflection (d), known as separation (S), referred to the initial position (z_0) and deflection (d_0), *i.e.*, the separation at the contact point (S_0):

$$\delta = (z-d) - (z_0 - d_0) = S - S_0$$

Substituting it in the preceding equation leads to:

$$F^{2/3} = \left[\frac{4E_y R^{1/2}}{3(1-\nu^2)}\right]^{2/3} \cdot (S - S_0) = \left[\frac{4E_y R^{\frac{1}{2}}}{3(1-\nu^2)}\right]^{\frac{2}{3}} \cdot S - \left[\frac{4E_y R^{\frac{1}{2}}}{3(1-\nu^2)}\right]^{\frac{2}{3}} \cdot S_0 = m \cdot S + K$$

where *m* is the slope of the function and *K* the intersection point of the function with the vertical axis. Thus,

$$K = -m \cdot S_0 \quad \rightarrow \quad S_0 = -\frac{K}{m}$$

In conclusion, the contact point, or the value of the separation at the point of contact, is calculated by dividing the intersection point of the linear function with the vertical axis and its slope.
